# The goo that binds us: how field resonance solves neuroscience’s binding and criticality problems

**DOI:** 10.3389/fncom.2026.1738326

**Published:** 2026-06-24

**Authors:** Tam Hunt

**Affiliations:** University of California, Santa Barbara, Santa Barbara, CA, United States

**Keywords:** binding problem, consciousness, criticality, cross-frequency coupling, EEG origins, electromagnetic fields, EM field hypothesis, energy modulation

## Abstract

The binding problem and the criticality problem represent two of neuroscience’s most persistent challenges: how do distributed neural processes create unified conscious experience, and how does the brain maintain optimal information processing at the critical boundary between order and chaos? This paper argues that both problems emerge from neuroscience’s “prickly” bias toward discrete, computational approaches and dissolve when we embrace “gooey” electromagnetic field perspectives. Drawing on Alan Watts’ philosophical dichotomy between “prickles” (precise, chopped-up particles) and “goo” (softer, continuous waves), I demonstrate how the EM field hypothesis provides natural solutions to spatial and temporal binding through cross-frequency coupling while simultaneously explaining neural criticality through volumetric field propagation. New evidence reveals that electromagnetic fields can entrain neural spike timing at thresholds as low as 0.74 mV/mm, establishing causal field-to-neuron communication. The 5,000-fold speed advantage of ephaptic field propagation (50 km/s vs. 10–100 m/s for spikes) enables rapid integration necessary for unified consciousness, while the volumetric nature of field propagation naturally generates the power-law scaling and critical avalanche dynamics observed across neural systems. Rather than requiring evolutionarily unstable fine-tuning of synaptic weights, criticality arises spontaneously from multi-scale electromagnetic field interactions. The implications extend beyond solving two technical problems to recognizing that cognition and consciousness are probably more gooey than prickly—not made ultimately of discrete computational events but continuous electromagnetic field dynamics produced by the brain and body.

## 1 Introduction: the binding problem as neuroscience’s tough nut to crack

In his philosophical writings, Alan Watts articulated a fundamental dichotomy that cuts to the heart of how we understand reality: “There are basically two kinds of philosophy. One’s called Prickles, the other’s called Goo. And prickly people are precise, rigorous, logical. They like everything chopped up and clear. Goo people like it vague. For example, in physics, prickly people believe that the ultimate constituents of matter are particles. Goo people believe it’s waves.” I would not say I like things “vague,” but I will tip my hand early in this paper: I’m going to make the case for goo over prickles for a new understanding of cognition and consciousness.

This distinction illuminates a deep divide in neuroscience that has profound implications for understanding consciousness. Neuroscience has overwhelmingly embraced the prickly approach, focusing obsessively on discrete neural spikes, precise synaptic connections, and chopped-up computational modules. This bias has led to remarkable advances in understanding neural mechanisms, but it has also created seemingly intractable problems–chief among them the binding problem.

The binding problem emerges directly from prevailing assumptions about consciousness. If consciousness consists of separate processing streams operating independently—one for color, another for motion, yet another for shape—then how do these streams combine to create unified conscious experience? The problem becomes even more acute when we consider that these processing streams operate at different speeds, in different brain regions, and with different temporal dynamics. How does the brain achieve the precise timing necessary to bind together different activities in the brain and body?

The alternative (gooey) perspective suggests a rather different approach. From this perspective, the binding problem resolves readily because the electromagnetic fields that constitute conscious experience operate as naturally integrated/coupled phenomena within the medium of the brain and body. Table 1 summarizes various aspects and implications of these contrasting approaches to thinking about cognition and consciousness.

**Table 1:** Prickly vs. gooey approaches to neuroscience and consciousness, an overview.

Aspect	Prickly thinking	Gooey thinking
Fundamental nature	Reality consists of discrete particles	Reality consists of continuous waves/fields; particles are highly concentrated standing waves
Core metaphor	Brain as digital computer	Brain as electromagnetic field medium
Primary substrate	Neural spikes and synaptic connections	Electromagnetic fields and resonance
Information Coding	Discrete spike trains (binary/digital)	Continuous field dynamics (analog)
Binding mechanism	Requires special binding circuits/convergence zones	Natural integration through field coherence
Communication speed	Limited by synaptic delays (10–100 m/s)	Field propagation at ~50,000 m/s
Information capacity	Linear, sequential processing	Volumetric (3D) parallel processing
Temporal binding	Complex synchronization mechanisms needed	Automatic through field coherence
Criticality maintenance	Active tuning of synaptic weights	Arises from multi-scale field interactions
1/f noise origin	Requires fine-tuning	Natural consequence of field dynamics
Neural avalanches	Sequential synaptic cascades	Electromagnetic resonance cascades
Energy efficiency	High metabolic cost for binding circuits	Minimal energy via resonance
Unity of consciousness	Paradoxical - how to bind separate modules?	Natural—fields are inherently unified
Measurement focus	Spike counts, firing rates	Field potentials, oscillations, phase
Consciousness theory	Emerges from computation	IS the electromagnetic field patterns
Philosophical position	Reductionist, mechanistic	Holistic, continuous

The binding problem represents a convergence of several challenges that have puzzled neuroscientists for decades. Early observations of ephaptic (field) interactions between neurons (Katz and Schmitt, 1940; Arvanitaki, 1942) were largely dismissed in favor of synaptic mechanisms.” Spatial binding concerns how features processed in different brain regions are integrated into unified percepts. Temporal binding addresses how events occurring at different times are experienced as simultaneous (Engel and Singer, 2001). Feature binding examines how different attributes of objects (color, shape, motion) are combined into unified representations (Treisman and Gelade, 1980). Cross-modal binding investigates how information from different sensory systems creates integrated experiences, as demonstrated in phenomena like the rubber hand illusion (Botvinick and Cohen, 1998).

Traditional approaches to these challenges have relied on increasingly complex computational theories involving convergence zones, timing mechanisms, oscillatory binding (von der Malsburg, 1981; Singer and Gray, 1995), and attention-based selection (Felleman and Van Essen, 1991; Mesulam, 1998; Felleman and Van Essen, 1991; Mesulam, 1998). However, these solutions face fundamental limitations. Convergence zones require anatomical structures that often do not exist. Timing mechanisms struggle with the variable delays inherent in neural processing. Oscillatory binding cannot account for the flexibility and context-sensitivity of conscious experience. Attention-based theories cannot explain how binding occurs even when attention is divided or absent.

The electromagnetic field hypothesis (Pockett, 2000, 2012; McFadden, 2002, 2020; Jones, 2013; Hunt and Schooler, 2019; Hunt and Jones, 2023) offers a rather different approach. Instead of requiring special mechanisms to bind together separately processed features, electromagnetic field patterns can simultaneously represent multiple features through overlapping wave patterns that interact through resonance and interference across large distances at high speeds. The speed of electromagnetic field propagation [47–57 km/s through brain tissue, Ruffini et al. (2018, 2020)] provides the temporal headroom necessary for complex cognition and consciousness, while the volumetric nature of field effects enables natural spatial integration across brain regions.

The emphasis on neural spike patterns as the basis of brain function is not arbitrary—it reflects compelling evidence and practical success (Crick and Koch, 1990; Humphries, 2020). Neural firing clearly controls motor output: specific spike patterns reliably trigger muscle contractions (Giszter et al., 2007). Similarly, sensory neurons fire in direct response to stimuli, with spike rates encoding stimulus intensity (Rieke et al., 1999). Decades of single-unit recording have revealed systematic relationships between spike patterns and behavior (Hebb, 1949; Shadlen and Newsome, 1998), creating an impressive edifice of spike-based neuroscience.

Given this success, why propose, as an alternative, field primacy for cognition and consciousness? Three considerations suggest a more nuanced picture than standard neuroscience offers:

First, correlation v. causation: The correlation between neural firing and many cognitive functions may still reflect fields as the primary causal agent, with spikes serving to maintain and modulate field dynamics rather than compute or integrate directly (Pockett, 2012; McFadden, 2020; Hales and Ericson, 2022). Evidence traditionally interpreted as “neural firing causes X” can in many cases equally support “fields cause X, and firing correlates because it shapes those fields” (Hunt and Jones, 2023). The recent demonstration that subthreshold field effects can entrain neural firing at 0.74 mV/mm (Lee et al., 2024; Lee and Koch, 2024) suggests bidirectional causation, with fields potentially upstream of some firing patterns.

Second, the evolutionary division of labor: Motor control and sensory processing may represent the original evolutionary function of neural firing—a function it continues to perform admirably (Hartline and Colman, 2007). These systems require precise, localized control: contract this muscle, detect that photon. Spike timing and rate coding excel at such tasks (Rieke et al., 1999). But higher cognition and unified conscious experience require something different: rapid integration across distributed brain regions, flexible binding of arbitrary features, and the creation of coherent phenomenal fields. The ~5,000-fold speed advantage of electromagnetic field propagation over even myelinated axons (Nunez, 2000; Nunez and Srinivasan, 2006, 2014), combined with volumetric processing capabilities, may explain why evolution co-opted field effects for these integration-demanding functions.

Third, levels of complexity: Simple organisms with limited nervous systems may indeed operate primarily through spike-based mechanisms. But as nervous systems scaled up, the binding and integration problems became acute. Our hypothesis is that electromagnetic field effects—particularly ephaptic coupling modulated by myelination patterns—provided the solution that enabled the explosion of cognitive complexity in vertebrates and especially mammals (Hunt and Schooler, 2019; Hunt, 2020; Hunt and Jones, 2023). Consciousness and higher cognition may have only evolved to their current sophistication once evolution “discovered” how to harness field effects systematically.

This perspective does not in any way dismiss spike-based neuroscience; it recontextualizes it (Hunt and Jones, 2023). Spikes may remain crucial for understanding motor control, sensory transduction, and maintaining the neural infrastructure that generates fields. But for the hard problems of consciousness, binding, and rapid whole-brain integration, the EM field dynamics that arise from collective neural activity may be doing the primary computational work, with spike patterns serving as both causes and consequences of those field dynamics in a complex feedback loop.

The explanatory gap for standard neuroscience remains profound. As neuroscientist Mark Humphries candidly admits in his comprehensive overview of spike-based neuroscience in a book called *The Spike*: “The most obvious chasm in our understanding is in all the things we did not meet on our journey from your eye to your hand [in terms of how spikes control these phenomena]. All the things of the mind I’ve not been able to tell you about, because we know so little of what spikes do to make them” (Humphries, 2020, p. 248). This acknowledgment—from a leading proponent of spike-based neuroscience—suggests that the traditional approach may be limited in addressing cognition and consciousness, even as it succeeds at explaining sensory transduction and motor control.

Another challenge is that standard neuroscience tools and paradigms were largely developed to study spikes rather than fields (Koch et al., 2016; Koch, 2020). We measure what we can measure, and spikes are often easier to isolate and analyze than mesoscopic field dynamics. The recent technical advances enabling measurement of weak endogenous fields (Lee et al., 2024; Ruffini et al., 2018, 2020; Cunha et al., 2024) are now allowing us to test these alternatives directly rather than inferring everything from spike patterns alone. It is an interesting time for neuroscience.

## 2 The strong evidence for electromagnetic primacy

### 2.1 Fields entraining neurons

One of the most significant developments in consciousness research in recent years has been the demonstration that electromagnetic fields can directly entrain neural activity at remarkably low thresholds. Lee et al. (2024) provided groundbreaking evidence that electric fields as weak as 0.74 mV/mm can causally entrain neural spike timing, establishing genuine field-to-neuron communication rather than merely correlational relationships. This represents direct empirical evidence for field-to-neuron causation, not simply neuron-to-field correlation, establishing causal field-to-neuron communication at the heart of neural processing.

This finding shifts our understanding of the relationship between electromagnetic fields and neural activity. Previous research had demonstrated correlations between field activity and consciousness, but skeptics could argue that fields were merely epiphenomenal byproducts of neural firing. The demonstration of causal field effects eliminates this objection, proving that electromagnetic fields can actively influence neural processing rather than simply reflecting it.

The threshold for field entrainment (0.74 mV/mm) falls well within the range of endogenous brain activity, as measured during normal cognitive tasks. This means that the brain naturally generates electromagnetic fields strong enough to entrain neural firing, suggesting that field effects operate continuously during normal brain function rather than representing exotic phenomena that emerge only under special circumstances.

The implications extend far beyond technical details about field strength. If electromagnetic fields can entrain neural firing at physiological thresholds, then the traditional hierarchy between neural activity and field effects must be reconsidered. Rather than neural firing driving field activity, the relationship appears bidirectional or even field-dominant. This supports what I call the strong electromagnetic field hypothesis: consciousness arises from field dynamics, while neural firing serves primarily as an energy source for maintaining and modulating these fields. The weaker version of this hypothesis, that EM fields can and do modulate neural firing and thereby play a role in cognition and consciousness, is explored in depth in Hunt and Jones (2023).

### 2.2 Distinguishing neural firing from electromagnetic field dynamics

A crucial distinction often obscured in neuroscience research concerns the difference between neural firing patterns and the electromagnetic field dynamics those firing patterns generate. Neural firing—the rate at which neurons generate action potentials—operates on millisecond timescales with frequencies typically ranging from 1 to 200 Hz. However, the electromagnetic fields produced by this neural activity can exhibit far richer dynamics, with oscillations spanning from ultra-slow frequencies (< 0.01 Hz) to very high frequencies potentially extending into the kilohertz range and beyond.

This distinction parallels the tree metaphor introduced earlier: while the trunk and branches (neural firing) oscillate on relatively slow timescales when buffeted by wind, the leaves and finest twigs (electromagnetic fields) can oscillate orders of magnitude faster. The bulk of information relevant to consciousness may reside in these rapid field dynamics, with neural firing providing the energetic substrate that maintains the field structure.

Recent work on parameterizing neural power spectra (Donoghue et al., 2020) has revealed that both periodic (oscillatory) and aperiodic (broadband) components contribute to electromagnetic activity measured via EEG and ECoG. The aperiodic component—characterized by 1/f power-law scaling—reflects the overall electromagnetic field environment within which oscillatory patterns are embedded. Building on foundational work on aperiodic neural activity (Buzsáki et al., 2012; Miller et al., 2009), Brake et al. (2024) demonstrated that aperiodic exponents measured during resting states could predict cognitive decline over 10 years, with the aperiodic component reflecting the fundamental balance between excitatory and inhibitory neural activity.

However, the predictive power of the aperiodic component should not be interpreted as diminishing the importance of oscillatory dynamics for consciousness. Rather, within the hypothesis explored in this paper, the aperiodic background provides the energetic foundation—the “power grid”—within which specific oscillatory patterns carry the detailed information necessary for conscious experience. The oscillations themselves, particularly their cross-frequency coupling relationships (Section 2.3), enable the rapid binding and integration that consciousness requires.

Recent ECoG studies reveal that broadband power increases, rather than narrow-band oscillatory patterns, provide strong correlates of cognitive processing (Manning et al., 2009). This broadband activity likely reflects the overall electromagnetic field strength within brain regions—the total energetic capacity available for field computations. But within this broadband envelope, specific oscillatory patterns at multiple frequencies interact through resonance and cross-frequency coupling to create the detailed structure of conscious experience.

Understanding what EEG signals represent remains a fundamental problem (Freeman, 2000, 2004; Cohen, 2017). The electromagnetic field dynamics recorded via EEG and other techniques reflect not merely the averaged firing of neuronal populations but the genuine field environment that Freeman (2000, 2004) identified as central to neural dynamics. The aperiodic baseline and the oscillatory patterns it supports are different but closely related electromagnetic phenomena, and they serve different functional roles: the aperiodic component reflects the overall field capacity as energetic source, while the oscillatory structure carries the specific information patterns that constitute complex cognition and consciousness.

### 2.3 Cross-frequency coupling: the field orchestra

Cross-frequency coupling represents one of the most compelling pieces of evidence for electromagnetic field theories of consciousness. The observation that brain oscillations at different frequencies interact through phase-amplitude coupling, frequency–frequency coupling, and other complex relationships suggests that consciousness arises from the orchestrated interaction of multiple electromagnetic field patterns operating simultaneously.

Cross-frequency coupling has been extensively studied as a mechanism for neural communication (Buzsáki and Draguhn, 2004; Lisman and Jensen, 2013). Canolty and Knight (2010) demonstrated that cross-frequency coupling increases during cognitive tasks requiring conscious processing, while remaining minimal during unconscious states. This pattern suggests that consciousness requires the coordinated interaction of electromagnetic fields operating at different temporal scales, from slow rhythms that coordinate large-scale brain networks to fast oscillations that enable local processing. Field disruption in pathological states such as epilepsy (Blumenfeld, 2012) demonstrates the necessity of coherent field patterns for normal consciousness.

The mathematics of cross-frequency coupling reveal natural mechanisms for information integration across temporal scales. Low-frequency oscillations can modulate the amplitude of high-frequency activity, enabling slow processes to influence fast computations. Conversely, high-frequency patterns can influence the phase of slow oscillations, allowing local processing to affect global coordination. These bidirectional influences create dynamic information integration that operates naturally through electromagnetic field interactions.

## 3 How coevolved field-infrastructure architecture binds us

Michael Levin’s groundbreaking research on the bioelectric code provides crucial evolutionary and conceptual grounding for electromagnetic field theories of consciousness. Levin has demonstrated that bioelectric patterns function as an ancient computational medium, enabling cells to encode, store, and propagate anatomical information across multiple spatial and temporal scales through what he terms “pattern memory”—stable electrical configurations that guide morphogenetic processes and can be modified by experience while persisting across cellular generations (Levin et al., 2018; Levin, 2023). This bioelectric infrastructure predates neural consciousness by hundreds of millions of years, establishing the computational foundation that consciousness would later co-opt for rapid integration across distributed neural networks. Critically, Levin’s distinction between rapid neural networks optimized for immediate behavioral control and slower bioelectric networks specialized for morphogenetic pattern maintenance parallels the distinction between fast myelinated pathways and slower unmyelinated field-based processing that our framework identifies as essential for consciousness. Moreover, his demonstration of hardware-software independence—where bioelectric patterns achieve consistent outcomes across different ion channel configurations—provides theoretical grounding for understanding consciousness as electromagnetic field dynamics that are enabled by, but not reducible to, the neural structures generating them (Levin, 2011). The binding and criticality problems that plague spike-based theories of consciousness dissolve when viewed through Levin’s bioelectric lens: pattern memory through electrical fields naturally enables the volumetric integration required for binding, while multi-scale bioelectric networks inherently exhibit the scale-free dynamics characteristic of criticality, without requiring the fine-tuned synaptic parameters that evolution could not reliably maintain. This perspective aligns with philosophical arguments that physicalism entails some form of panpsychism (Strawson, 2006).

The binding paradox emerges from the logical contradiction inherent in discrete computational approaches to consciousness. If consciousness consists of separate processing modules operating independently (Zeki, 1993, 2003), then consciousness itself becomes impossible—there would be no unified subject to experience the outputs of these modules. The binding problem is thus not a technical challenge but a logical impossibility generated by prickly assumptions.

Consider the visual system’s traditional decomposition into separate processing streams for color, motion, and form. Each stream operates independently with different processing speeds, anatomical pathways, and computational characteristics. The color system processes information relatively slowly through the parvocellular pathway, while motion detection operates rapidly through the magnocellular system, with areas such as MT (Born and Bradley, 2005) specialized for motion processing. Form processing involves complex interactions between multiple cortical areas with varying delays. If consciousness required binding these separate streams together, the timing challenges alone would be insurmountable.

The electromagnetic field solution eliminates this paradox through natural superposition properties. Electromagnetic fields from different processing regions naturally overlap and interfere within the brain’s conducting medium, creating integrated field patterns that simultaneously represent multiple features. Rather than requiring separate color, motion, and form representations that must somehow be combined, integrated field patterns contain information about all features simultaneously through wave interference patterns. Somatosensory regions developed field characteristics suitable for spatial processing while remaining integrated with motor areas for action planning. The entire brain operates as a unified field medium with regional specializations that enhance rather than fragment processing.

### 3.1 Spatial binding through volumetric integration

Spatial binding represents perhaps the most straightforward demonstration of electromagnetic field advantages over computational approaches. Traditional theories require anatomical convergence zone (Felleman and Van Essen, 1991; Mesulam, 1998; Felleman and Van Essen, 1991; Mesulam, 1998) where information from different brain regions physically meets, but such convergence zone (Felleman and Van Essen, 1991; Mesulam, 1998; Felleman and Van Essen, 1991; Mesulam, 1998) often do not exist or cannot account for the flexibility of spatial binding.

Electromagnetic fields solve spatial binding through volumetric integration that operates across the entire brain volume simultaneously. Field patterns generated in occipital visual areas naturally propagate through the conducting medium of brain tissue, interacting with fields from temporal, parietal, and frontal regions. This creates integrated field patterns that span multiple brain regions without requiring anatomical convergence points.

The speed of electromagnetic field propagation (47–57 km/s) enables near-instantaneous integration across brain volume. A field pattern generated in primary visual cortex reaches frontal regions in approximately 4 milliseconds, faster than any synaptic transmission chain could achieve. This temporal advantage is crucial for consciousness, which requires integration across brain regions within the time frame of conscious experience.

### 3.2 Temporal binding through field dynamics

Temporal binding poses even greater challenges for computational approaches than spatial binding. Neural processing involves variable delays ranging from milliseconds for local processing to hundreds of milliseconds for complex cognitive operations. If consciousness required precise timing coordination across these variable delays, the computational complexity would be overwhelming.

Traditional solutions involve oscillatory mechanisms that allegedly coordinate timing across brain regions through synchronized neural firing. However, these mechanisms face fundamental limitations. Oscillatory periods are typically much longer than the temporal precision required for consciousness. Phase relationships are disrupted by the variable delays inherent in neural processing. Most critically, oscillatory mechanisms cannot account for the temporal flexibility of conscious experience.

Electromagnetic field dynamics provide natural solutions to temporal binding through the speed advantages of field propagation and the coevolved infrastructure that coordinates timing. The 5,000-fold speed advantage of ephaptic transmission over neural spikes provides temporal headroom that enables consciousness to integrate information across the variable delays of neural processing.

Field propagation at 47–57 km/s means that electromagnetic signals traverse the entire brain volume in approximately 4 microseconds (ms). This creates a temporal reference frame within which the variable delays of neural processing become negligible. Information that arrives at different brain regions at different times can be integrated within field patterns that maintain temporal coherence across the entire brain.

### 3.3 Feature binding through superposition

Feature binding represents the classic formulation of the binding problem: how are different attributes of objects (color, shape, motion, texture) combined into unified object representations? Computational approaches require complex mechanisms for associating features that were processed independently, often involving attention-based selection or temporal correlation.

Electromagnetic fields solve feature binding through natural superposition properties that eliminate the need for special binding mechanisms. When electromagnetic fields from different feature processing regions overlap in brain tissue, they create interference patterns that simultaneously contain information about all features. Rather than requiring separate color, shape, and motion representations that must be artificially combined, integrated field patterns represent all features simultaneously.

The mathematics of electromagnetic superposition provide precise mechanisms for feature integration. Constructive interference enhances field patterns where features are consistent, while destructive interference reduces patterns where features conflict. This creates natural selection mechanisms that favor coherent feature combinations while suppressing inconsistent alternatives.

Feature binding operates across multiple spatial scales simultaneously. Local field integration within cortical columns binds features processed by nearby neurons, while global field patterns integrate features across distant brain regions. This multi-scale integration enables both fine-grained feature associations and global object representations within the same electromagnetic framework.

The temporal dynamics of feature binding reflect the interaction between fast local processing and slow global integration. High-frequency field oscillations enable rapid feature detection within specialized regions, while low-frequency patterns coordinate feature integration across regions. Cross-frequency coupling naturally arises from these multi-scale dynamics, creating the temporal patterns observed during object recognition tasks.

### 3.4 Cross-modal binding through unified field architecture

Cross-modal binding presents particularly challenging problems for computational approaches because it requires integration across sensory systems that operate with fundamentally different temporal dynamics, spatial organizations, and computational principles. Visual processing emphasizes spatial relationships, auditory processing focuses on temporal patterns, and somatosensory processing involves both spatial and temporal components.

Traditional approaches require convergence zone (Felleman and Van Essen, 1991; Mesulam, 1998; Felleman and Van Essen, 1991; Mesulam, 1998) where information from different sensory systems physically meets, such as the superior temporal sulcus for audiovisual integration. However, these anatomical solutions cannot account for the flexibility and context-sensitivity of cross-modal binding, nor can they explain how cross-modal effects emerge even when convergence zones (Felleman and Van Essen, 1991; Mesulam, 1998; Felleman and Van Essen, 1991; Mesulam, 1998) are damaged.

Electromagnetic fields solve cross-modal binding through the unified field architecture that spans all sensory systems. Field patterns generated by visual, auditory, and somatosensory processing naturally interact within the brain’s conducting medium, creating integrated patterns that simultaneously represent information from multiple modalities. This eliminates the need for special convergence zone (Felleman and Van Essen, 1991; Mesulam, 1998; Felleman and Van Essen, 1991; Mesulam, 1998) or binding mechanisms.

The speed advantages of electromagnetic field propagation enable cross-modal integration within the time frame of conscious experience. Traditional explanations for cross-modal effects invoke lengthy synaptic transmission chains that cannot account for the rapid integration observed behaviorally. Field-based integration operates at speeds compatible with conscious experience, enabling genuine real-time cross-modal binding.

## 4 The electromagnetic speed revolution

### 4.1 Quantifying the speed differential

The speed differential between electromagnetic field propagation and neural transmission represents one of the most compelling arguments for the electromagnetic field hypothesis. Ruffini et al. (2018, 2020) demonstrated that ephaptic field effects propagate at speeds of approximately 47 km/s in gray matter and 57 km/s in white matter, compared to 0.1–120 m/s for neural transmission. This represents a linear speed advantage of approximately 500–5,000 times in favor of electromagnetic transmission.

However, this linear speed advantage dramatically understates the true information processing potential of electromagnetic fields. The volumetric nature of field propagation means that information capacity scales as the cube of linear dimension, while the three-dimensional interference patterns enable parallel processing that scales exponentially with field complexity. When these factors are combined with the speed advantage, the total information processing advantage reaches potentially billions of times greater than neural spike transmission.

The mathematical relationship underlying this advantage can be expressed as: Information Capacity = Speed × Volume × Parallelism × Interference Patterns. Each component provides substantial advantages for electromagnetic fields over neural spikes, and their multiplication yields the exponential scaling that enables consciousness.

Understanding the 50 km/s propagation speed.

The 50 km/s propagation speed cited from Ruffini et al. (2018, 2020) requires careful explanation, as it represents diffusive field propagation in brain tissue rather than electromagnetic wave propagation in the classical sense. At neural frequencies (< 1 kHz), brain tissue conductivity (*σ* ≈ 0.3 S/m) and high relative permittivity (εr ≈ 10^6) create a regime where the conduction term dominates over the displacement current in Maxwell’s equations.

The wave equation for electric fields in conductive media is:

$$\nabla^{2}E-\mu \sigma (\partial E/\partial t)-\mu \varepsilon (\partial^{2}E/\partial t^{2})=0.$$

The relative importance of the two time-derivative terms depends on frequency. For brain tissue at typical neural frequencies (10–100 Hz), the ratio of conduction to displacement current is approximately 60,000:1, meaning the conduction term dominates completely. The wave equation thus reduces to a diffusion equation:

$$\nabla^{2}E\approx \mu \sigma (\partial E/\partial t).$$

The effective propagation speed in this diffusive regime is:

$$v_{\mathrm{eff}}=\surd (2\omega /\mu \sigma ).$$

For a 50 Hz signal in gray matter (σ ≈ 0.3 S/m), this yields approximately 46 km/s, close to Ruffini et al.’s detailed calculations. This is fundamentally different from electromagnetic wave propagation in low-conductivity media like saline, where EM waves propagate at approximately *c*/9 ≈ 33,000 km/s.

While pure diffusion equations mathematically exhibit infinite propagation speed, the finite propagation speed observed by Ruffini et al. (2018, 2020) arises from the physical constraints of neural tissue. Ruffini’s finite-element models incorporate nonlinear boundary conditions at neural membranes, capacitive effects at cell boundaries, and the discrete cellular architecture of brain tissue. These factors create an effective reaction–diffusion system where the “reaction” terms represent current sources/sinks at neural membranes. The resulting propagation speed represents not pure diffusive spread but rather the coordinated progression of field perturbations constrained by the tissue’s electrical properties and cellular structure. This finite speed emerges from the coupling between extracellular field dynamics and membrane currents, creating a self-consistent propagation pattern rather than unbounded diffusion.

Crucially, the diffusive character of field propagation is not a limitation but precisely what enables global integration. Unlike discrete information channels, diffusion-like propagation naturally integrates across space through superposition, allowing for genuine spatial binding rather than point-to-point information transfer. Moreover, Ruffini et al. demonstrate that this speed increases approximately 10-fold at higher frequencies achieved in gamma band and above, meaning that faster oscillations may propagate even more rapidly through neural tissue.

While 50 km/s is much slower than light-speed EM propagation, it remains 50,000 × faster than chemical synaptic transmission (~1 m/s) and 500 × faster than the fastest axonal transmission (~100 m/s). At this speed, field effects can span the entire human brain (~15 cm diameter) in approximately 3 milliseconds—orders of magnitude faster than typical neural processing timescales, enabling true spatial integration across distributed neural populations within each cycle of neural oscillations (Table 2).

**Table 2:** Speed differences in field transmission and computation (Ruffini et al., 2018, 2020; Anastassiou et al., 2011; Hartline and Colman, 2007; Hunt and Schooler, 2019; Nunez and Srinivasan, 2006; Tasaki, 1953).

Parameter	Synaptic transmission	Ephaptic field propagation	Advantage factor
Linear speed	10–100 m/s (unmyelinated)	47,000–57,000 m/s	~500–5,000×
Linear speed	100–120 m/s (myelinated)	47,000–57,000 m/s	~400–500×
Volumetric processing	1D (linear pathways)	3D (field propagation)	10^4^–10^9^×
Whole-brain traversal	200–2000 ms	<5 ms	40–400×
Energy per operation	~10^−15^ J	~10^−18^ J	~1,000 × more efficient
Information density	Sequential, discrete	Parallel, continuous	~125 billion×
Frequency range	0.1–200 Hz	0.1 Hz-1 THz	~10,000×

### 4.2 Information density: beyond bits to field patterns

Traditional information theory, based on discrete bits, cannot capture the information processing capabilities of electromagnetic fields. Field patterns can represent information through amplitude, frequency, phase, and spatial distribution simultaneously, creating information densities that far exceed digital approaches.

Recent calculations suggest that electromagnetic field patterns in brain tissue could theoretically process information at rates exceeding 10^15 operations per second, compared to approximately 10^11 operations per second for high-end digital processors. This advantage emerges from the parallel processing capabilities of field dynamics, where all points within a coherent volume can process information simultaneously.

Evolution harnessed this information density through regional specialization. Different brain areas evolved field characteristics optimized for different types of information processing—some regions optimized for high-frequency temporal processing, others for complex spatial patterns, still others for cross-frequency integration. This specialization enables the brain to exploit the full information density potential of electromagnetic fields.

Field-based information processing operates through fundamentally different principles than digital computation. Rather than sequential logical operations, field patterns evolve according to physical laws that enable massive parallel processing. Interference patterns naturally implement complex computational operations, while resonance effects provide selection mechanisms that enhance relevant information while suppressing noise.

### 4.3 Energy efficiency: the metabolic argument

Perhaps the most compelling argument for electromagnetic field primacy comes from metabolic considerations. The human brain consumes approximately 20% of the body’s total energy despite representing only 2% of body weight. This extraordinary energy consumption suggests that neural processing involves fundamental inefficiencies that electromagnetic field processing might overcome.

Electromagnetic field processing operates through resonance mechanisms that require minimal energy input once established. Like tuning forks that can maintain oscillations with periodic small energy inputs, brain field patterns can maintain complex dynamics with relatively modest metabolic support. This explains how consciousness can operate continuously despite the brain’s limited energy budget.

Field-based processing also enables energy efficiency through natural integration mechanisms that eliminate the need for complex binding circuits. Rather than requiring separate neural circuits to bind together independently processed features, field superposition achieves integration naturally without additional energy costs. This represents a fundamental architectural advantage that evolution discovered through the coevolution of fields and infrastructure.

### 4.4 How goo maintains criticality

The brain maintains itself at criticality—poised at the edge of chaos where information processing is optimized (Beggs and Plenz, 2003; Beggs, 2008; Plenz and Thiagarajan, 2007; Shew et al., 2011; Shew and Plenz, 2013; Timme et al., 2016). This critical state manifests as neural avalanches: cascading patterns of activity that follow power-law distributions across multiple scales. However, current models relying solely on synaptic transmission cannot explain a fundamental puzzle: avalanches appear to propagate at speeds that far exceed what sequential synaptic transmission permits (Chiang et al., 2019).

I propose that neural avalanches are not primarily synaptic phenomena but electromagnetic resonance cascades—rapid propagation of coherent activity through the brain’s continuous field dynamics. This perspective reveals criticality not as something the brain must actively maintain through complex feedback mechanisms, but as the natural consequence of multi-scale electromagnetic field interactions. This approach goes beyond brain dynamics. The interorgan resonance framework (Young et al., 2022) extends field-based integration beyond the brain to include heart and gastric systems.

When a neural population fires, it generates a local field perturbation that propagates at approximately 47–57 km/s through neural tissue (Ruffini et al., 2018, 2020)—nearly 500 times faster than myelinated axon conduction and 5,000 times faster than unmyelinated fibers. This perturbation does not simply decay with distance; it resonates with the intrinsic oscillatory dynamics of neighboring neural populations, potentially triggering their activation if they are near threshold.

The critical insight is that these field-mediated cascades follow different physics than synaptic avalanches. While synaptic transmission requires sequential, point-to-point propagation constrained by axonal conduction velocities and synaptic delays, electromagnetic resonance cascades can leap across cortical space, recruiting distant populations through field coherence. A resonance cascade can traverse the entire cortical sheet in under 5 milliseconds—faster than a single synaptic transmission across just a few neurons.

This speed differential becomes even more dramatic when we consider volumetric propagation. Synaptic connections follow linear pathways, but electromagnetic fields propagate in three dimensions. The information-carrying capacity scales as the cube of the linear propagation advantage, yielding potential efficiency gains of 10^8^ to 10^9^ over purely synaptic mechanisms. Evolution could not ignore such massive advantages in information processing capacity.

Power law scaling in neural systems—including 1/f noise in brain recordings and avalanche size distributions—indicates criticality and scale-free dynamics across multiple scales. However, as Zakharov et al. (2012) have demonstrated in their work on wave turbulence, power law decay is characteristic of non-linear dynamical systems more generally and does not by itself require high-speed dynamics.

The 1/f noise ubiquitous in brain recordings—where power spectral density scales inversely with frequency—arises naturally from multi-scale field interactions. The nonlinear interactions between different frequency components of neural fields can generate power law scaling through mechanisms similar to those in turbulent systems. The brain may exhibit a form of wave turbulence in which electromagnetic field modes interact nonlinearly, transferring energy across scales.

Field-based integration provides a biophysically plausible mechanism for achieving and maintaining criticality across multiple spatial and temporal scales. Consider the superposition of resonant modes across scales:

S(f)=Σk∣Ak(f)∣2∝f^(−β).

where β ≈ 1 at criticality. This pink noise signature does not require fine-tuning or active maintenance; it arises spontaneously when electromagnetic fields interact across the multiple spatial and temporal scales present in neural tissue.

The brain’s nested oscillatory hierarchy—from ultra-slow fluctuations (0.01 Hz) through delta, theta, alpha, beta, and into high gamma (>100 Hz)—creates a natural substrate for critical dynamics. Each frequency band can trigger resonance cascades at its characteristic scale, with cross-frequency coupling enabling cascade propagation across scales. The power laws of neural avalanches may reflect not carefully balanced synaptic weights but the inherent multi-scale resonance properties of electromagnetic fields in conductive neural media.

The connection being made is not that speed → power laws, but rather that field physics provides the mechanism enabling scale-free integration across the whole brain at very rapid speeds. The speed of field propagation enables critical dynamics to span the entire brain within behaviorally relevant timescales, allowing what would otherwise be local critical patches to participate in unified brain-wide criticality.

Laukkonen and colleagues have identified brain dynamics that appear “paradoxically” fast—coordination patterns that seem to exceed the speed limits of synaptic transmission (Laukkonen and Chandaria, 2025). Recent proposals invoke turbulence-like dynamics as a potential explanation, suggesting that the statistical properties of turbulent systems enable rapid information transfer without requiring direct field propagation (Deco and Kringelbach, 2020).

While turbulent dynamics certainly characterize brain activity, I suggest that turbulence itself emerges from the underlying electromagnetic substrate. The vortices and eddies observed in neural dynamics—analogous to those in fluid turbulence—represent patterns in the brain’s electromagnetic field rather than purely synaptic phenomena. Turbulent mixing achieves its remarkable efficiency precisely because it occurs in a continuous medium where information can propagate through field dynamics rather than requiring discrete synaptic steps.

The “paradox” dissolves when we recognize that the brain operates through two complementary timescales: slow synaptic/metabolic processes that maintain the energetic substrate (the “power grid”), and fast electromagnetic dynamics that perform the actual computation (the “information highway”). Neurotransmitter systems act as field-controlled rheostats, rapidly shifting local brain regions toward supercritical states (enhanced processing) or subcritical states (stability) based on computational demands.

Several experimental observations support field-mediated criticality over purely synaptic mechanisms:

Non-synaptic propagation: Chiang et al. (2019) and Chiang and Durand (2020) demonstrated that slow periodic activity in hippocampal slices can self-propagate in the complete absence of synaptic transmission, with propagation characteristics consistent with ephaptic coupling. These avalanche-like events follow power-law distributions indistinguishable from those observed with intact synaptic transmission.

Speed mismatches: High-density microelectrode recordings reveal avalanche propagation velocities that exceed predictions from synaptic models by 1–2 orders of magnitude, particularly for long-range cascade events (Rubinov et al., 2011). The observed velocities match predictions from electromagnetic field propagation.

Field precedence: Local field potential changes consistently precede spiking activity during avalanche initiation by 2–5 milliseconds—the opposite of what synaptic models predict but exactly what field-mediated cascades require (Fröhlich and McCormick, 2010).

Transcranial perturbation: Weak electric fields applied transcranially can trigger or suppress avalanche dynamics at field strengths (0.5–1 V/m) far below the threshold for direct neuronal activation, demonstrating that fields causally influence cascade dynamics (Reato et al., 2010).

Understanding neural avalanches as electromagnetic resonance cascades provides unexpected insight into the binding problem. Each avalanche represents a transient binding event—a momentary coherence across distributed neural populations that creates unified perceptual or cognitive states. The power-law distribution of avalanche sizes explains why consciousness exhibits both stability and flexibility: small avalanches maintain local processing while occasional large avalanches enable global integration.

The critical brain is thus not merely optimized for information processing in some abstract sense; it is precisely tuned to enable the rapid, flexible binding operations that consciousness requires. The same gooey field dynamics that solve the binding problem also maintain the brain at criticality. Rather than being separate puzzles requiring independent solutions, binding and criticality are complementary aspects of the brain’s electromagnetic nature.

### 4.5 Temporal dynamics of field integration

A critical question about field-based integration concerns temporal dynamics: how do field effects operating at microsecond timescales relate to neural firing occurring on millisecond timescales? At first glance, this might seem paradoxical—if field propagation is orders of magnitude faster than neural firing, how can fields causally influence the “basic dynamic process of the brain”?

The key insight is that field effects and neural firing operate in parallel rather than competitively. Field propagation at 50 km/s means the entire brain can be integrated within ~3 ms—far faster than typical interspike intervals (10–100 ms) and well within the temporal window of single spike cycles for most neurons. This enables spatial binding to occur continuously during ongoing neural activity, solving the binding problem through rapid whole-brain integration.

Recent work by Lee and Koch (2024) demonstrates that endogenous electric fields can entrain neural firing within 1–2 ms, showing these effects operate on timescales entirely compatible with neural computation. The field provides real-time spatial integration of information already present in distributed spike trains. Moreover, the cross-frequency coupling hierarchy enables integration across multiple timescales simultaneously: theta rhythms (~125–250 ms period) provide the temporal envelope within which gamma oscillations (~10–33 ms period) unfold, and field integration occurs fast enough (~3 ms) to maintain phase coherence across this entire hierarchy.

### 4.6 A hypothesis and a tree metaphor

The hypothesis explored in this paper goes further, however, than viewing fields as merely modulatory or parallel systems. The ephaptic field systems—suggests that electromagnetic fields emerging from the brain’s neuroanatomy—may constitute a distinct causal structure that exists above and beyond the neuroanatomy itself, and carry far more information. While neural firing operates on millisecond timescales, the ephaptic field structure supported by that firing can interact on microsecond timescales. Since these fields interact far faster than neural firing, this system is a causal structure that can carry significantly more information than neural firing alone.

Using a tree metaphor: while the trunk and larger branches might oscillate on the scale of seconds when buffeted by winds, the twigs and leaves may oscillate on the scale of microseconds, thus containing far more information than the trunk and branches. In the brain’s EM field structure, there exists a system of nested hierarchies exhibiting cross-frequency coupling that allows for fine-tuned information transfer across the system in a way that may not rely on the underlying neuroanatomy except as a supporting substrate.

To extend the metaphor: if the tree’s leaves were connected by very thin fibers (spider webs perhaps), a small breeze on one side of the tree could disturb leaves on the other side even if the trunk or branches did not move at all. This represents field-to-field interactions that can occur independently of changes in the underlying neural firing patterns—the bulk of information relevant to cognition and consciousness may reside in the fast field dynamics (the “twigs and leaves”), with neural firing providing the structural and metabolic support (the “trunk and branches”).

### 4.7 Biophysical mechanisms and field magnitudes

Understanding whether electromagnetic fields play a functionally significant role in cognition and consciousness requires careful consideration of field magnitudes, spatial scales, and coupling strengths at realistic physiological parameters. Recent computational and experimental work provides strong evidence that ephaptic coupling at realistic physiological scales can support functionally relevant dynamics.

#### 4.7.1 Computational validation at realistic scales

Ruffini et al. (2018, 2020) provided detailed compartmental models with physiologically accurate conductivities (gray matter: 0.276 S/m; white matter: 0.126 S/m), relative permittivities (~10^6 at low frequencies), and realistic neuronal geometries. They demonstrated that endogenous field magnitudes of 1–5 mV/mm are sufficient to modulate spike timing by 1–3 milliseconds—highly significant given the temporal precision of neural codes. These effects are sustained and cumulative across populations, meaning that even “weak” coupling at the single-neuron level can have strong effects at the network level.

Pinotsis et al. (2016) and Pinotsis and Miller (2023) took a different approach, using mean-field models to demonstrate that ephaptic coupling enables memory network formation through field-mediated plasticity. Their work shows that field effects are not “diffuse” or “non-specific” but can support functionally specific neural representations. *In vivo* field magnitudes (1–10 mV/mm measured empirically) are sufficient to bias synaptic plasticity through field-mediated depolarization. Different memory patterns create distinct field configurations that can be stably maintained through feedback between fields and synaptic weights.

#### 4.7.2 Experimental evidence

Anastassiou et al. (2011) and Anastassiou and Koch (2015) performed careful *in vitro* experiments in rat hippocampal slices, demonstrating that endogenous fields of 1–5 mV/mm—well within the range measured *in vivo*—reliably modulate spike timing by 1–3 milliseconds. These effects depend on neuronal morphology and field orientation, as predicted by cable theory and computational models. Field effects are sufficiently strong to influence network dynamics, including synchronization and oscillatory patterns, despite the “weak” coupling (few percent modulation of firing rate).

Lee and Koch (2024) demonstrated cell-class-specific field entrainment in mouse cortex, showing that different interneuron subtypes respond differently to endogenous fields based on their biophysical properties. This suggests that field effects can provide selective modulation of specific circuit elements—far from “non-specific.”

#### 4.7.3 Functional specificity

The functional specificity for cognition and consciousness comes from multiple mechanisms.

Cross-frequency coupling creates temporal structure across resonance chains, with slow oscillations modulating the amplitude of fast rhythms in nested hierarchies (Canolty and Knight, 2010).

Harmonic relationships between frequency bands enable selective coupling between specific neural populations while excluding others. Precise integer ratios (1:2, 1:3, etc.) allow stable phase relationships to be maintained over time (Klimesch, 2012, 2018).

Phase relationships carry information, with harmonic frequency arrangements facilitating cognitive performance (Rodriguez-Larios and Alaerts, 2019; Rodriguez-Larios et al., 2020), about functional coordination that can be rapidly modulated through field effects. The relative phase between oscillations in different regions indicates their functional interaction patterns (Fries, 2005, 2015).

Cell-type-specific responses depend on morphology and biophysical properties, providing another layer of functional selectivity beyond spatial patterns in the field itself. As Lee and Koch (2024) showed, the same field can have different effects on different cell types. Hales and Ericson (2022) argue that electromagnetic field effects provide the critical bridge between micro- and macroscale neural dynamics.

The key insight is that fields do not need to provide point-to-point information transmission (which axons already do) but rather enable spatial integration across distributed populations. The “intricacy” comes from temporal patterning, frequency relationships, and cell-type-specific responses rather than complex spatial patterns in the field itself. Hales and Ericson (2022) argue that electromagnetic field effects provide the critical bridge between micro- and macroscale neural dynamics.

## 5 Implications for neuroscience research

### 5.1 Beyond spike counting

Traditional approaches that focus on neural spike patterns while ignoring field dynamics may be missing the primary substrate of conscious experience. Future research must simultaneously measure field dynamics and infrastructure characteristics across multiple spatial and temporal scales. This requires new technologies that can capture the interaction between electromagnetic fields and myelinated architecture during conscious processing. Current approaches that study these phenomena separately cannot reveal their integrated operation.

Much existing neuroscience research may need reinterpretation from a field-infrastructure perspective. Studies that attributed cognitive functions to specific brain regions may have actually identified specialized field computation zones within the larger electromagnetic architecture of consciousness.

The timing relationships observed in neural recording studies may reflect field coordination rather than synaptic transmission delays. Many phenomena attributed to synaptic plasticity may actually involve changes in field characteristics that alter information processing capabilities.

Pharmacological studies may need reinterpretation in terms of field effects rather than neurotransmitter actions. Many drugs that affect consciousness may work by altering field characteristics or field-infrastructure coordination rather than through direct synaptic effects.

### 5.2 New experimental approaches

The field-infrastructure framework suggests novel experimental approaches that could definitively test electromagnetic theories of consciousness. Experiments that manipulate field patterns while monitoring consciousness could establish causal relationships between field dynamics and conscious experience.

Developmental studies that track myelination and field characteristics simultaneously could reveal how consciousness arises from field-infrastructure coordination. These studies could identify critical periods where field computation capabilities develop and test how experience shapes this development.

Comparative studies across species could illuminate universal principles of consciousness architecture. These studies could test whether consciousness capabilities correlate with field-infrastructure coordination rather than simple brain size or neural number.

Intervention studies using electromagnetic stimulation could test whether restoring normal field patterns can improve consciousness in various disorders. These studies could provide both therapeutic benefits and fundamental insights into consciousness mechanisms.

## 6 Conclusion

The solution to each aspect of the binding problem follows naturally from the properties of electromagnetic fields produced by the brain and body, revealing why traditional approaches have failed for decades while the field solution was always present in the electromagnetic dynamics of consciousness. A formal name for this is field computing, a kind of analog computing, as opposed to the purported digital (or quasi digital) computing of neural firing.

Spatial binding dissolves as a problem when we recognize that electromagnetic field waves naturally create unified information spaces across brain regions through volumetric integration. Rather than requiring anatomical convergence zone (Felleman and Van Essen, 1991; Mesulam, 1998; Felleman and Van Essen, 1991; Mesulam, 1998) where separated processing streams meet, overlapping field patterns create continuous integration that preserves spatial relationships while enabling flexible coordination.

Temporal binding becomes trivial when electromagnetic field propagation at 47–57 km/s provides the temporal headroom necessary for consciousness, while myelinated infrastructure coordinates timing across regions. Rather than struggling to overcome synaptic delays through complex timing mechanisms, field-based coordination achieves near-instantaneous integration across the brain’s volume.

Feature binding arises naturally from the superposition properties of electromagnetic fields operating within specialized but integrated field architecture. Rather than requiring special mechanisms to combine separately processed features, electromagnetic field patterns simultaneously represent multiple features through overlapping wave patterns that interact through resonance and interference. Different brain regions evolved specialized field characteristics for different features while maintaining integration through shared electromagnetic dynamics.

Cross-modal binding occurs automatically through field patterns that naturally span multiple sensory modalities within the unified field architecture. Rather than requiring complex convergence mechanisms, field patterns enable direct interaction between different sensory systems.

The solution to the binding problem has implications that extend beyond neuroscience to fundamental questions about the nature of mind and reality. For neuroscience, the shift from prickly spike-counting to gooey field dynamics represents a substantial change. Rather than focusing on discrete neural events, neuroscience must embrace the continuous electromagnetic field dynamics that provide the natural substrate for consciousness.

For consciousness studies, the binding problem was intractable for prickly approaches because consciousness cannot be decomposed into discrete components—it seems to be irreducibly analog, not digital.

For artificial intelligence, the recognition that binding requires field-like mechanisms operating within sophisticated infrastructure suggests that artificial consciousness may not be achievable through purely computational approaches, at least not based on current von Neumann computing architectures. AI systems that aspire to consciousness may need to incorporate EM field nested hierarchical architectures that enable the flexible, context-sensitive integration that biological consciousness exhibits.

Electromagnetic continuity connects us to the fields that permeate the universe while maintaining our individuality through the specialized infrastructure that shapes our specific field characteristics. We exist as semi-stable local patterns in the universal electromagnetic field.

## Data Availability

The original contributions presented in the study are included in the article/supplementary material, further inquiries can be directed to the corresponding author/s.
